# Pulmonary Vein Occlusion and Lung Infarction after Radiofrequency Ablation of Atrial Fibrillation

**DOI:** 10.1155/2020/2357846

**Published:** 2020-07-27

**Authors:** Julyan Al Fori, Maryam Al Belushi, Mohammed Al Shuraiqi, Ghalia Al Mohanny, Rashid Al Umairi, Nasser Al Busaidi

**Affiliations:** ^1^Department of Chest Medicine, The Royal Hospital, Muscat, Oman; ^2^Department of Cardiology, The Royal Hospital, Muscat, Oman; ^3^Department of Radiology, The Royal Hospital, Muscat, Oman

## Abstract

**Background:**

Pulmonary vein (PV) radiofrequency ablation (RFA) is an effective technique for a selected group of patients with atrial fibrillation (AF) refractory to antiarrhythmic drugs (Alfudhili et al., 2017). However, pulmonary vein occlusion is a potentially rare, sometimes severe, complication which may present clinically as nonspecific respiratory symptoms, signifying pulmonary vein stenosis, that are often underrecognized or misdiagnosed, leading to progression of the low-grade stenosis to complete occlusion if not treated with timely intervention (Alfudhili et al., 2017). *Case Presentation*. We report the first case of haemoptysis, three months postradiofrequency ablation (i.e., late complication) secondary to pulmonary vein occlusion that was diagnosed by computed tomography angiogram (CTA), which showed occlusion of 2 out of 4 native pulmonary veins.

**Conclusion:**

The cause of haemoptysis in this patient was pulmonary vein occlusion, secondary to radiofrequency ablation, as demonstrated in the CTA.

## 1. Case Report

A 33-year-old male presented with refractory AF, nine months postradiofrequency ablation. The patient developed symptomatic palpitations due to atrial fibrillation, and he was prescribed flecainide and bisoprolol. Despite the antiarrhythmic therapy, the patient continued to have significant symptomatic atrial fibrillation. Hence, the patient was taken for a second RFA.

The preablation echocardiogram demonstrated normal ventricular function and pulmonary pressures. During the RFA procedure, the left atrium and all four PVs were individually isolated and mapped using the CARTO-3 advanced 3D cardiac mapping system. It was noticed that the patient has left common pulmonary veins. Then, voltage mapping was done revealing a connection between the superior pulmonary veins and the anterior wall of the atrium. Antral ablation lesions were done, electrical isolation was created and performed with energy (35-40 watts) applied on both superior veins, and complete pulmonary vein isolation was achieved and verified by pacing attempts and verification of exit block in both veins. Remapping by the voltage map was done to assure complete isolation between the veins and the Lt.atrium, and it confirmed complete isolation.

Echocardiography done post procedure showed no pericardial effusion. After the curative ablation therapy, the patient was kept on aspirin and rivaroxaban to prevent AF recurrence as well as pulmonary venous and/or arterial thrombosis.

**Figure 1:**
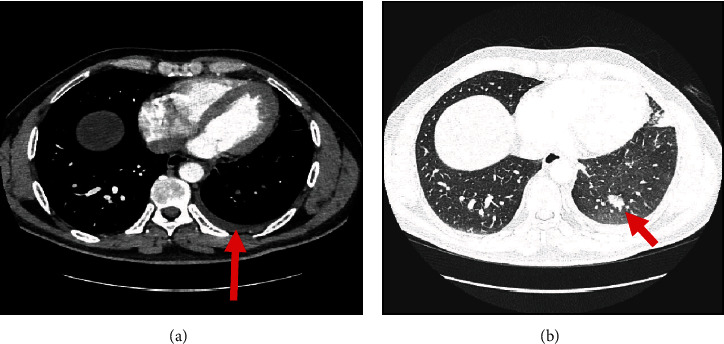
Computed tomography: (a) left pleural effusion (arrow); (b) consolidation and GGO in LUL (arrow). GGO: ground glass opacity; LUL: left upper lobe.

**Figure 2:**
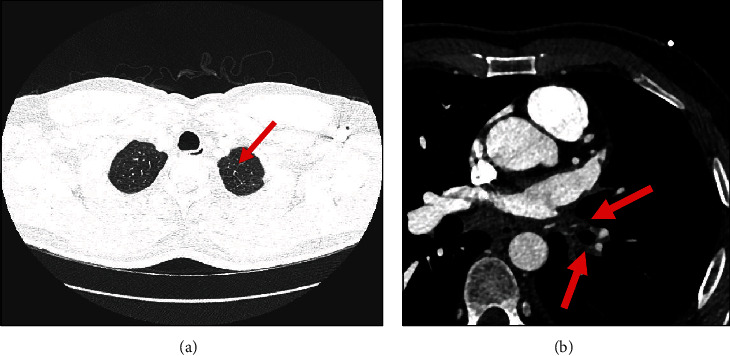
CTPA: (a) unilateral pulmonary edema with interlobular septal thickening (arrow); (b) Lt.PVsO (arrow). CTPA: computed tomography pulmonary angiogram; Lt.PVsO: left pulmonary vein occlusion.

**Figure 3:**
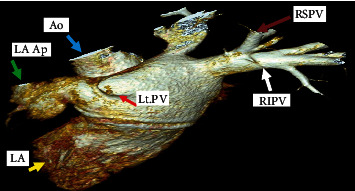
Three-dimensional cardiac CT reconstruction images: absent of left pulmonary veins following AF ablation (red arrow). CT: computed tomography; Ao: aorta (blue arrow); LA Ap: left atrial appendage (green arrow); LA: left atrium (yellow arrow); RSPV: right superior pulmonary vein (brown arrow); RIPV: right inferior pulmonary vein (white arrow); AF: atrial fibrillation.

**Figure 4:**
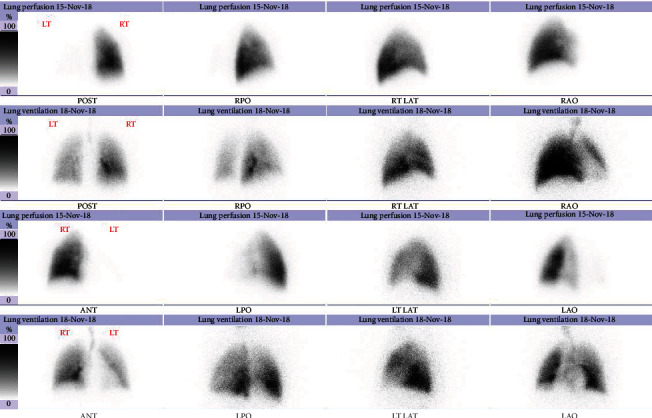
Absent perfusion in the left lung field in all perfusion views; in ventilation images, there is adequate ventilation of the left lung which is lower compared to the right lung.

However, two months later, the patient presented to the emergency department with cough associated with haemoptysis and mild left-sided pleuritic chest pain. He underwent computed tomography pulmonary angiogram (CTPA), which showed multiple patches of consolidation as well as ground glass opacities in the left upper lobe and lingula with minimal left pleural effusion, as shown in Figures 1(a) and 1(b); differentials were either an infective process or lung infarction. The patient was given broad spectrum antibiotics and referred to the pulmonology clinic for further investigation. Meanwhile, he was seen in cardiology OPD for follow-up, and he was advised to stop rivaroxaban as his CHA2DS2-VASc score was noted to be zero, in the setting of active haemoptysis. However, despite this, his haemoptysis persisted. Therefore, a contrast-enhanced CTPA was performed that showed features of unilateral left pulmonary venous congestion, and the left superior and inferior pulmonary veins were not visualized (as shown in Figures 2(a) and 2(b)), both signifying occlusion, most likely due to the prior ablation. In addition to that, he also underwent a coronary CTA which revealed that the right superior and inferior pulmonary veins are well-opacified with contrast; however, both left superior and inferior pulmonary veins are not visualized in the arterial phase (as shown in Figure 3). These findings of coronary CTA were consistent with the CTPA finding of left pulmonary vein occlusion. The patient also had a lung perfusion study, which showed absent perfusion in the left lung field in all perfusion views. In the ventilation images, there was adequate ventilation of the left lung, though it was lower compared to the right lung (as shown in Figure 4). A bronchoscopy was done as well and reported to be normal (no bleeding).

## 2. Discussion

AF is one of the most common forms of cardiac arrhythmias and represents a major cause of stroke. The prevalence of AF increases with age; in people older than 65 years, it reaches up to 5%. Over 90% of ectopic beats that cause AF arise in the pulmonary veins and nearly 50% of them originate in left superior pulmonary veins [1].

Catheter-based radiofrequency ablation for AF is an effective treatment for patients with symptomatic AF refractory to therapy with antiarrhythmic drugs and has been increasingly performed worldwide [2]. The frequency of PV occlusion, a well-recognized complication following an AF ablation of pulmonary veins, has been declining due to the improvement of technique and operator experience [3]. Pulmonary vein occlusion is defined as more than 95% stenosis or complete loss of patency of a pulmonary vein as seen on chest CT [4]. The incidence rates vary from 1.3% to 15.6% in the reported articles, but the true incidence of PV occlusion following an AF radiofrequency ablation is still controversial [5]. The pathophysiology of progression of the injury in time was demonstrated in animal data showing intimal proliferation, collagen replacement of necrotic myocardium, endovascular contraction, and proliferation of the elastic lamina [6]. Both the arterial systems and pulmonary venous are well known to be interrelated. It has been shown that sudden occlusion of a pulmonary vein is soon followed by gradual decrease and then cessation of the arterial flow to the affected segment, and this is related to a decline in the arteriovenous gradient as well as compression by the developing tissue edema. Consequently, the involved alveoli are affected by the resulting ischemia and tissue edema, leading to atelectasis, infarction, or infection [7].

PV occlusion acquired after AF ablation varies in severity from asymptomatic to nonspecific symptoms, including persistent cough, haemoptysis, shortness of breath, and pleuritic chest pain [3]. Symptoms of PV occlusion depend on many factors, such as the number of veins involved, the time course of disease, and the severity of stenosis. Almost all patients with mild (<50%) or moderate PVS (50–70%) have no symptoms [1].

Chest X-ray finding can be nonspecific such as pulmonary and interstitial opacities and pleural effusion, so the final diagnosis is obtained with transoesophageal echocardiogram, magnetic resonance imaging, or CTPA after injection of intravenous contrast in the late phase to reduce flow artifacts [8].

PV occlusion can be treated acutely with balloon dilatation of the PV, although the long-term outcome is doubtful. Thus, treatment options are limited currently, and restenosis after PV intervention has been described and considered relatively frequent [9]. In the limited cases with irreversible occlusion with significant symptoms, dilation and stenting can be ineffective, and eventually, they require lung resection [10]. Hence, patients with PV occlusion need careful assessment and follow-up because of the risk of recurrence, which occurs in 50% of patients within 1 year [9]. Prevention of PV occlusion is mainly related to placing the ablation site from inside to outside the orifice of the PV and reducing the ablation temperature and energy applied [9]. This strategy helps to decrease the risk of PV occlusion to less than 1% [9].

**Figure 5:**
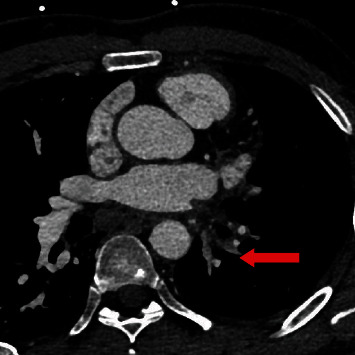
Left PV post balloon angioplasty (red arrow).

Our patient was treated initially as pneumonia, based on his symptoms and imaging findings; however, he showed no improvement in the haemoptysis. He had CTPA which showed complete occlusion of the left superior and inferior pulmonary veins. Later, he underwent bronchoscopy which showed no active bleeding, and bronchial washing was negative for bacterial growth. Ventilation/perfusion scan showed no perfusion in whole left lung. Based on the diagnosis of left pulmonary vein occlusion in CTPA, the patient underwent balloon angioplasty of the same as shown in Figure 5; the procedure was complicated by arrhythmia and pericardial effusion which was drained successfully. Repeated ECHO showed normal ventricular function with no pericardial effusion.
